# Household Food Insecurity Is Associated With Higher Adiposity Over Time Among Adolescents in Louisiana

**DOI:** 10.1111/ijpo.70084

**Published:** 2026-02-05

**Authors:** Ashley Fenton, Amanda E. Staiano, Michael Celestin, Tekeda Ferguson, Candice A. Myers, Tung‐Sung Tseng, Stephanie T. Broyles

**Affiliations:** ^1^ School of Public Health Louisiana State University Health Sciences Center New Orleans Louisiana USA; ^2^ Pennington Biomedical Research Center Louisiana State University Baton Rouge Louisiana USA

**Keywords:** adiposity, adolescents, food insecurity, longitudinal study, obesity

## Abstract

**Background:**

Few studies have examined how household food insecurity may impact longitudinal changes in adiposity among adolescents.

**Objective:**

We investigated the link between household food insecurity and 2‐year change in adolescent adiposity, with sex as a potential moderator.

**Methods:**

Analyses included 222 adolescents living in and around Baton Rouge, Louisiana, who participated in the TIGER Kids study (baseline: June 2016–December 2017; follow‐up: January 2018–August 2019). Household food security was measured using a validated two‐question parent‐reported survey. Adiposity outcomes were collected using anthropometry, dual‐energy X‐ray absorptiometry and abdominal magnetic resonance imaging (MRI). Multivariable multilevel models assessed associations between household food security and changes in adiposity.

**Results:**

At baseline, the participants were 12.9 ± 1.9 years, 50.5% female, 37.4% non‐White or Hispanic, 31.5% had obesity, and 11.3% were food insecure. Food‐insecure adolescents exhibited significantly greater increases in BMI_p95_ (*b* = 6.0% ± 2.2%, *p* = 0.0082), waist circumference (*b* = 4.1 ± 1.7 cm, *p* = 0.0158), total body fat percentage (b = 3.0% ± 1.3%, *p* = 0.0194) and visceral adipose tissue mass (*b* = 0.16 ± 0.06 kg, *p* = 0.0163), compared to their food‐secure peers. The effect of food insecurity on adiposity did not differ between boys and girls.

**Conclusions:**

This longitudinal study highlights the deleterious influence of food insecurity on adolescent adiposity. Efforts to alleviate food insecurity may play an important role in preventing obesity in adolescents.

## 1 Introduction

Obesity prevalence within the U.S. has been consistently increasing since at least 2000 [1]. According to the National Health and Nutrition Examination Survey (NHANES) conducted from 2017 to March 2020 (pre‐pandemic), 22.2% of U.S. adolescents aged 12–19 had obesity [2], indicating a nearly two percentage point increase in obesity prevalence from the 2015–2016 NHANES [1, 2]. National guidelines have emphasized the importance of early identification, prevention and treatment of obesity, as its consequences manifest early and escalate over time [3, 4, 5]. Adolescents with obesity are more likely to suffer social and psychological consequences (e.g., low self‐esteem, depressive symptoms) [6], remain obese into adulthood, face a higher risk of developing cardiometabolic diseases and experience other severe health problems (e.g., hypertension) throughout their lives [7].

An issue that paradoxically occurs alongside obesity, food insecurity refers to insufficient food for a healthy and active life and is a growing problem in the U.S. population [8, 9]. In 2023, food insecurity affected 13.5% of all U.S. households and 17.9% of households with children [8]. While food insecurity is most commonly linked to poor food quality and disrupted eating patterns [10, 11, 12], like obesity, it also has social and psychological consequences for adolescents [13, 14]. For example, adolescents experiencing food insecurity are at a significantly higher risk of experiencing depressive symptoms, anxiety, behavioral problems and suicidal ideation compared to their food‐secure peers [13, 14, 15].

While a significant amount of research connects food insecurity to obesity risk in adults [16], the evidence regarding its relationship with adolescent adiposity remains inconsistent [12, 15]. A recent meta‐review of the consequences of food insecurity for children and adolescents concluded that evidence for associations between food insecurity and weight status was mixed and that these associations may differ by age and sex [15]. This meta‐review further highlighted the need for rigorous longitudinal studies to address knowledge gaps. Noteworthy, one of the included reviews that focused solely on longitudinal associations between food insecurity and obesity remarked on the absence of longitudinal studies involving adolescents [12]. Furthermore, none of the longitudinal studies included outcomes beyond those derived from BMI. In response to these recognised needs, the current study focused on youth aged 10–18 and investigated the association between household food insecurity and changes in four comprehensive, objective measures of adiposity over a 2‐year period. We hypothesized that food‐insecure adolescents would experience larger 2‐year increases in adiposity compared to adolescents from food‐secure households. We further explored whether the food insecurity–adiposity relationship differed between boys and girls.

## 2 Methods

### 2.1 Study Participants

This analysis used data from the Translational Investigation of Growth and Everyday Routines in Kids (TIGER Kids; NCT02784509) study, a comprehensive prospective cohort study conducted among adolescents living in and around Baton Rouge, Louisiana. The goal of TIGER Kids was to understand the factors influencing childhood obesity and related health outcomes. Participants were recruited through various channels, including previous study participants, local schools, community groups, and targeted social media advertisements in the Baton Rouge area. Inclusion criteria for the TIGER Kids study consisted of being 10–16 years old, having a body weight of less than 500 pounds due to equipment limitations, and having the ability to understand and complete study procedures. Multiple adolescents from the same household were allowed to participate in the study. Adolescents were excluded if they were pregnant, were on restrictive diets, had limited mobility, or had cognitive/language barriers. The complete eligibility criteria and a detailed study description have been published elsewhere [17].

Before enrollment, adolescents provided written informed assent, and caregivers provided written informed consent. The Pennington Biomedical Research Center's Institutional Review Board reviewed and approved the study protocol to ensure ethical compliance and participant safety (IRB# 2016‐028‐PBRC).

TIGER Kids participants underwent evaluations during baseline and follow‐up visits (approximately 2 years after the baseline visit) at the Pennington Biomedical clinic. Baseline data collection occurred between June 2016 and December 2017, and follow‐up visits occurred between January 2018 and August 2019. At an orientation visit, participants and caregivers provided assent/consent, received detailed information about the study, and were given an accelerometer with instructions on how to wear it over the week following the visit. Within 3 weeks of the orientation visit, participants returned for their baseline visit to return the accelerometer and complete all other study procedures, including interviews, questionnaires, physical examinations, and laboratory tests. Study visits were conducted year‐round, including over school holidays and the summer. Baseline and follow‐up assessments were conducted at the same time of the year whenever feasible to strive for seasonal and scheduling consistency. Follow‐up visits tracked longitudinal changes by repeating the accelerometry, dietary intake, anthropometric, and imaging procedures [3, 4]. Data were managed using REDCap (Research Electronic Data Capture), a secure web‐based application for research data capture [18, 19].

**FIGURE 1:**
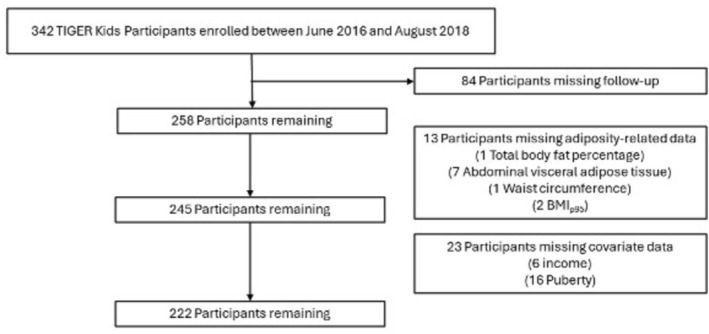
CONSORT diagram for the analytic sample for the present study.

Of the 342 participants enrolled in the original study, the current analytic sample comprised 222 participants (64.9% of the full sample) nested within 176 households, after exclusions for missing or incomplete data. Specifically, 129 participants were excluded based on not attending the follow‐up visit (*n* = 84) or missing adiposity measures (*n* = 13). An additional 34 participants were excluded because of missing covariates retained in adjustment models, including 16 participants who were removed from the analysis because their self‐reported pubertal status showed backward progression that was deemed biologically implausible. See Figure 1. A sensitivity analysis was conducted that imputed values for individuals removed for missing adiposity or covariate data; this procedure is described below in ‘Statistical Analysis’.

### 2.2 Measurements

#### 2.2.1 Baseline Household Food Security Status

Baseline household food security status, the predictor variable, was assessed using the Hunger Vital Sign (HVS) survey, a parent‐reported, validated, two‐question survey created by the American Academy of Paediatrics based on the United States Department of Agriculture (USDA) Household Food Security Survey Module (HFSSM) [20]. Multiple studies across different populations and settings have shown the HVS to have high sensitivity and specificity in identifying food insecurity compared to the gold‐standard HFSSM [20, 21, 22, 23]. HVS questions include: (1) ‘Within the past 12 months, we worried whether our food would run out before we got money to buy more’; and (2) ‘Within the past 12 months, the food we bought just didn't last and we didn't have money to get more’ [20]. An affirmative answer to either question indicated household food insecurity. While household food security was also assessed at follow‐up, there were too few households changing food security status to describe adiposity changes across the various categories as has been done in other studies [12]. However, we conducted a sensitivity analysis with food insecurity pooled into a single variable that indicated food insecurity at either baseline or follow‐up.

#### 2.2.2 Anthropometric Measures

Height and weight measurements were taken following standardised procedures and converted into age‐ and sex‐specific BMI percentiles utilising national reference data [24, 25, 26]. Height was assessed with a Harpenden stadiometer (Holtain Limited, Crymych, UK) to the nearest 0.1 cm. Participants, barefoot, stood straight with their heels and backs aligned against the stadiometer. They inhaled and held their breath while the assessor carefully adjusted the height to align with the Frankfort Horizontal Plane for accurate measurement. Weight was recorded using a Michelli GSE 460 scale (G.T. Michelli Co., Baton Rouge, LA), with measurements taken twice and averaged to the nearest 0.1 kg. A third measurement was conducted if the two initial weight measures varied by over 0.5 units. Participants only wore hospital gowns and undergarments during this process. The percentage of the 95th percentile (BMI_p95_) was derived from the 2022 extended BMI‐for‐age growth charts based on the participants' age, height and weight [24, 25, 26]. BMI_p95_ is recommended for classifying weight status in adolescents with severe obesity since BMI *z*‐scores do not adequately correlate with adiposity measures for BMIs above the 97th percentile [27]. Waist circumference was recorded in centimetres at the natural waist with a non‐elastic tape measure, ensuring clothing was moved aside.

#### 2.2.3 Body Composition

Standard imaging and positioning protocol were utilised to evaluate total body fat mass. A whole‐body scan using a General Electric (GE) iDXA scanner (GE Medical Systems, Milwaukee, WI), which uses dual‐energy X‐ray absorptiometry (DXA) technology, was employed to estimate body fat. Encore (version 16.6; GE Medical Systems) for Windows automatically analysed scans. To determine the total body fat percentage, total fat mass (kg) was divided by body weight and multiplied by 100 (total fat mass/body weight × 100) [28].

A magnetic resonance imaging (MRI) system (Tesla's Electric Discovery 750w 3.0, GE Medical Systems, Milwaukee, WI) utilising the IDEAL‐IQ pulse sequence produced water‐only, fat‐only, in‐phase and out‐of‐phase images during a single acquisition that required a 20‐s breath‐hold [29]. This system evaluated the percentages and mass of abdominal visceral adipose tissue (VAT). The ANALYZE software package (CNSoftware, Rochester, MN) supported the image analysis. MRI scanning measured adipose tissue mass in kilograms. A skilled technician manually outlined the VAT on every fifth image, which was then used to estimate VAT mass in kilograms [28, 29].

### 2.3 Covariates

#### 2.3.1 Sociodemographic Characteristics

Demographic data were collected through parent‐reported surveys, including the child's race/ethnicity, sex, age, parental marital status and parental education levels, at the adolescent's first clinical visit. Race and ethnicity were merged into a single variable classified as ‘White non‐Hispanic’ and ‘non‐White or Hispanic’, as Black and Hispanic participants were too few for separate categorisation. For analysis, marital status and education were dichotomised into married versus unmarried and some college or less versus bachelor's degree or more, respectively. A continuous measure of poverty (ratio of household income to poverty) was calculated at the household level based on the 2017 federal poverty guidelines [30] using parent‐reported household income and household size; this measure was categorised for analysis as ≤ 200%, 201%–399%, and ≥ 400% of the 2017 federal poverty guidelines.

#### 2.3.2 Puberty

Participants self‐reported their pubertal development using standardised, validated images depicting Tanner Stages from 1 (pre‐pubertal) to 5 (post‐pubertal) for both boys and girls [31]. Puberty was categorised into pre/peri‐puberty and post‐puberty, and a variable was created to identify stage changes from baseline to follow‐up. Identifying pubertal status is important as adolescents progress rapidly through puberty, which can influence weight/adiposity gain depending on sex [32].

#### 2.3.3 Physical Activity

Physical activity was measured using ActiGraph GT3X+ tri‐axial accelerometers (ActiGraph, Fort Walton Beach, FL). Accelerometers were worn on the right‐mid‐axillary line, 24 h a day for 7 days, using an elasticised belt, yielding detailed data on movement patterns, intensity, and duration. Acceptable accelerometer data encompassed 4 days with a minimum of 10 h of active wear each day, including at least one weekend day. Activity intensity cutpoints were determined using a validated algorithm and evaluated based on the work of Evenson et al. [33] Counts per 15‐s epoch (CPE) are categorised as follows: 0–25 CPE indicates sedentary behaviour, 26–573 CPE denotes light physical activity, 574–1002 CPE represents moderate physical activity and 1003 CPE or higher signifies vigorous physical activity. The Evenson cutpoints [33] were selected because data were collected every 15 s, which more accurately captures the intermittent nature of physical activity in children and adolescents compared to cutpoints derived from 30‐ [34] or 60‐s [35] epochs. Wear‐time‐adjusted average daily minutes of moderate‐to‐vigorous physical activity (MVPA_adj_) were calculated by scaling individual MVPA minutes, the total of both moderate and vigorous activities, to the average wear time of 862 min. These MVPA_adj_ values were considered as a potential control variable in regression analyses.

### 2.4 Statistical Analysis

Descriptive statistics were computed to characterise the sample, providing central tendency and dispersion measures for all key variables. Multivariable multilevel (children nested within household) linear regression models (PROC MIXED) were used to assess the relationship between 2‐year changes in adiposity and food security status. Separate models were conducted for each adiposity measure, with the dependent variable being the 2‐year change in adiposity (the difference between baseline and follow‐up values).

All analyses included dichotomised household food security status (i.e., secure vs. insecure) and were adjusted for baseline adiposity. We created a parsimonious adjustment model across all adiposity outcomes as follows: (1) race/ethnicity, income‐to‐poverty ratio and pubertal stage changes were included in all models as these covariates were deemed important for face validity of the analyses. These covariates were retained unless *p* ≥ 0.9, where model stability would be compromised [36]. (2) Inclusion of other theoretically‐informed covariates, including maternal education, paternal education, baseline parent marital status, baseline MVPA and changes in MVPA, were assessed via backwards selection [36]. These covariates were retained where *p* < 0.9 across all models to ensure model stability and where *p* < 0.2 in at least two adiposity models; this criterion for retention is recommended for better control of confounding [36, 37]. As a result of this process, all analytic models were adjusted for baseline adiposity, race/ethnicity, income‐to‐poverty ratio, changes in pubertal stages and maternal education.

We also performed a second set of analyses, stratified on sex, informed by known differences in growth changes during puberty between boys and girls [38] and previous studies that have found differences in the effect of food insecurity on boys and girls [12]. These stratified models yielded estimates of the 2‐year adiposity changes and differences in these changes between food‐insecure and food‐secure households for boys and girls separately. For each adiposity outcome, the interaction term between food insecurity and sex in a single model that included both sexes was used to formally test for effect modification by sex on the food insecurity–adiposity relationship [39].

To assess whether results were sensitive to the removal of 36 individuals who attended their year 2 visit but who had missing data, we multiply‐imputed missing values using Markov chain Monte Carlo methods (PROC MI), conducted analyses of the imputed datasets as described above, and then summarised the results across the imputed datasets (PROC MIANALYZE).

All statistical analyses were conducted using SAS version 9.4 (Cary, NC), and statistical significance was accepted at *p* < 0.05.

## 3 Results

### 3.1 Study Population Characteristics

In our sample, the mean age of study participants at baseline was 12.9 years, and 37.4% identified as non‐White or Hispanic (Table 1). Overall, 11.2% of adolescents lived in food‐insecure households at baseline, and 24.3% of participants lived in households at or below 200% of poverty guidelines, with 9.5% living in poverty. Nearly a third (31.5%) had obesity (i.e., 95th BMI percentile), and 16.7% had severe obesity (i.e., 120% of the 95th BMI percentile). Across all measures of adiposity, baseline adiposity was higher for food‐insecure adolescents (Table 2).

**TABLE 1:** Characteristics of study participants, overall and by baseline household food security status (*n* = 222).

	Overall (*n* = 222)	Food secure (*n* = 197)	Food insecure (*n* = 25)	*p*
Baseline age (years), mean (SD)	12.9 (1.9)	12.9 (1.9)	13.3 (2.3)	0.2345
Race/ethnicity, *n* (%)				0.0412
White, non‐hispanic	139 (62.6)	128 (65.0)	11 (44.0)	
Non‐white or hispanic	83 (37.4)	69 (35.0)	14 (56.0)	
Sex, *n* (%)				0.0625
Boys	110 (49.5)	102 (51.8)	8 (32.0)	
Girls	112 (50.5)	95 (48.2)	17 (68.0)	
Pubertal status, *n* (%)				0.7211
Pre/peri‐pubertal at both time points	86 (38.7)	78 (39.6)	8 (32.0)	
Pre/peri‐ to post‐pubertal	69 (31.1)	61 (31.0)	8 (32.0)	
Post‐pubertal at both time points	67 (30.2)	58 (29.4)	9 (36.0)	
Baseline ratio of household income to poverty, *n* (%)				< 0.0001
≤ 200%	54 (24.3)	37 (18.8)	17 (68.0)	
201%–399%	54 (24.3)	48 (24.4)	6 (24.0)	
≥ 400%	114 (51.4)	112 (56.9)	2 (8.0)	
Parent's marital status, *n* (%)				< 0.0001
Married	130 (58.6)	123 (62.4)	7 (28.0)	
Divorced or separated	52 (23.4)	47 (23.9)	5 (20.0)	
Never married	38 (17.1)	27 (13.7)	11 (44.0)	
Widowed parent	2 (1.0)	0 (0.0)	2 (8.0)	
Mother education, *n* (%)				< 0.0001
High school diploma/GED or less	27 (12.2)	24 (12.2)	3 (12.0)	
Associate degree or 1–3 years of college	49 (22.1)	35 (17.8)	14 (56.0)	
Bachelor's degree	82 (36.9)	75 (38.1)	7 (28.0)	
Graduate/professional degree	64 (28.8)	63 (32.0)	1 (4.0)	
Father education, *n* (%)				< 0.0001
High school diploma/GED or less	64 (29.5)	48 (24.9)	16 (66.7)	
Associate degree or 1–3 years of college	48 (22.1)	41 (21.2)	7 (29.2)	
Bachelor's degree	61 (28.1)	60 (31.1)	1 (4.2)	
Graduate/professional degree	44 (20.3)	44 (22.8)	0 (0.0)	

**TABLE 2:** Adiposity of TIGER Kids participants, overall and by baseline household food security status.

	Overall (*n* = 222)	Food secure (*n* = 197)	Food insecure (*n* = 25)	*p*
Baseline adiposity, mean (SD)				
BMI_p95_ (%)	92.6 (28.7)	90.2 (27.3)	111.0 (32.7)	0.0006
Waist circumference (cm)	77.6 (18.5)	76.1 (17.5)	89.5 (22.1)	0.0005
Total body fat (%)	33.9 (10.4)	33.1 (10.1)	40.1 (11.5)	0.0015
Abdominal visceral adipose tissue (kg)	0.53 (0.46)	0.49 (0.40)	0.84 (0.74)	0.0303
Change in adiposity, mean (SD)				
BMI_p95_ (%)	2.2 (9.1)	1.6 (8.8)	6.4 (10.0)	0.0120
Waist circumference (cm)	4.6 (6.7)	4.3 (6.6)	6.8 (7.3)	0.0821
Total body fat (%)	−0.6 (5.2)	−0.9 (5.3)	1.3 (3.1)	0.0035
Abdominal visceral adipose tissue (kg)	0.09 (0.24)	0.07 (0.23)	0.20 (0.33)	0.0833

Abbreviation: BMI_p95_, percentage of the 95th BMI percentile.

### 3.2 Baseline Household Food Insecurity and Changes in Adiposity

Mixed effects models showed significant associations between baseline household food insecurity and changes in all adiposity measures (Table 3). Compared to food‐secure adolescents, those from food‐insecure households had higher 2‐year increases in BMI_p95_ (*b* = 6.0% ± 2.2%, *p* = 0.0082), waist circumference (*b* = 4.1 ± 1.7 cm, *p* = 0.0158), total body fat percentage (*b* = 3.0% ± 1.3%, *p* = 0.0194) and VAT (*b* = 0.16 ± 0.06 kg, *p* = 0.0163).

**TABLE 3:** Adjusted 2‐year changes in adiposity by food security status (*n* = 222).

Outcome	Food insecure	Food secure	Difference	*p*
BMI_p95_, %^a^	7.2 (2.0)**	1.2 (0.8)	**6.0 (2.2)**	0.0082
WC, cm^a^	8.0 (1.5)***	3.9 (0.6)***	**4.1 (1.7)**	0.0158
TBF, %^a^	1.8 (1.2)	−1.2 (0.5)**	**3.0 (1.3)**	0.0194
VAT, kg^a^	0.21 (0.06)**	0.06 (0.02)**	**0.16 (0.06)**	0.0163

*Note:* Bold values indicate statistical significance. *p < 0.05; **p < 0.01; ***p < 0.001.

Abbreviations: BMI_p95_, percentage of the 95th BMI percentile; TBF, total body fat percentage; VAT, abdominal visceral adipose tissue; WC, waist circumference.

^a^ Least square means [mean (SE)] from models adjusted for race/ethnicity, pubertal status across both time points, income‐to‐poverty ratio, and maternal education.

### 3.3 Household Food Insecurity and Changes in Adiposity for Boys and Girls

Among girls, no adiposity changes were significantly associated with household food insecurity, although differences approached significance (*p* < 0.1) for BMI_p95_, waist circumference, and VAT (Table 4). Among boys, the change in VAT was significantly associated with household food insecurity (0.25 ± 0.10 kg, *p* = 0.0150). Despite the general lack of statistical significance, for both boys and girls, there was a consistent pattern across all measures that food‐insecure adolescents experienced deleterious changes in adiposity larger in magnitude than their food‐secure counterparts. There was no evidence that food insecurity had different relationships with adiposity between boys and girls (all interaction *p*‐values > 0.33).

**TABLE 4:** Adjusted 2‐year changes in adiposity by food insecurity status and sex.

Outcome	Boys (*n* = 110)				Girls (*n* = 112)				Interaction *p* ^a^
	Food insecure	Food secure	Difference	*p*	Food insecure	Food secure	Difference	*p*	
BMI_p95_, %^b^	5.0 (3.2)	0.1 (1.1)	4.9 (3.4)	0.1565	8.1 (2.8)**	2.4 (1.2)	5.7 (3.1)	0.0676	0.6954
WC, cm^b^	7.5 (2.5)**	4.0 (0.8)***	3.6 (2.7)	0.1932	7.7 (1.9)***	4.1 (0.8)***	3.6 (2.1)	0.0899	0.8034
TBF, %^b^	−0.3 (2.1)	−3.5 (0.7)***	3.2 (2.3)	0.1699	2.5 (0.9)	0.6 (0.4)	1.9 (1.1)	0.0757	0.3355
VAT, kg^b^	0.28 (0.09)**	0.03 (0.03)	**0.25 (0.10)**	0.0150	0.15 (0.07)*	0.07 (0.03)*	0.08 (0.08)	0.2741	0.3265

*Note:* Bold values indicate statistical significance. *p < 0.05; **p < 0.01; ***p < 0.001.

Abbreviations: BMI_p95_, percentage of the 95th BMI percentile; TBF, total body fat percentage; VAT, abdominal visceral adipose tissue; WC, waist circumference.

^a^ *p*‐value for interaction term (food insecurity *x* sex effect in a single model; formal test for sex difference in the effect of food insecurity on adiposity outcome).

^b^ Least square means [mean (SE)] from models adjusted for race/ethnicity, pubertal status across both time points, income‐to‐poverty ratio, and maternal education.

### 3.4 Sensitivity Analyses

One sensitivity analysis pooled food insecurity into a single variable that indicated food insecurity at either baseline or follow‐up. Across all outcomes, 2‐year changes in adiposity were not different across adolescents from households food insecure at both time points (*n* = 12) and those food insecure at either baseline (*n* = 11) or follow‐up (*n* = 12) (BMI_p95_: *p* = 0.9102; WC: *p* = 0.9922; TBF: *p* = 0.5118; VAT: *p* = 0.7791). Likewise, results were similar to those presented in Table 3, with models showing significant associations between (pooled) household food insecurity and changes in all adiposity measures (Table S1). An additional analysis examined whether results were sensitive to the removal of 36 individuals with missing covariate (*n* = 23) or outcome (*n* = 13) values. In this sensitivity analysis, results were also consistent with those presented in Table 3 (Table S1).

## 4 Discussion

This study examined the relationship between baseline household food insecurity and changes in adolescent adiposity over 2 years. Our findings suggest that food insecurity is associated with larger increases in adiposity, reinforcing that economic hardship can contribute to adverse health outcomes in adolescents [1, 8]. Specifically, we found that food‐insecure adolescents exhibited significantly greater increases in BMI_p95_, waist circumference, total body fat percentage and VAT compared to their food‐secure counterparts. We also investigated whether the effect of food insecurity on adiposity differed between boys and girls and, across all adiposity measures, found no evidence that sex modifies this relationship.

Research investigating associations between food insecurity and adiposity in adolescents has produced mixed results [12, 15]. Among the three longitudinal studies in a recent systematic review that report results from samples that followed children into early adolescence, one reported no associations between food insecurity and obesity [40]; the second reported higher BMI *z*‐scores and risk of overweight and obesity for food‐insecure eighth graders [41]; and the third found that BMI increases from K‐8th grade were significantly higher for girls (not boys) from food‐insecure households [42]. Our study adds to this literature, finding that food insecurity was linked to significant increases in four adiposity measures over 2 years, in a sample that extends the age range included in these prior studies. Hence, our results indicate that the negative impact of food insecurity on weight‐related outcomes may continue across development stages. Additionally, our study showed this relationship across multiple objective measures of obesity, expanding on the BMI‐based measures reported in other studies.

With respect to differences between boys and girls, the systematic review by St. Pierre et al. [12] reported that three studies found associations between food insecurity and BMI/BMI *z*‐score for girls but not boys, while three other studies tested for interactions by sex and failed to find differences [15]. Our results align with prior studies that find no differences between boys and girls. We note, however, that the TIGER Kids study was not specifically powered to test for sex differences, and the number of food‐insecure adolescents was small, further limiting our power to test for interactions by sex. Additional research may be needed to reconcile findings and explore mechanisms that could contribute to sex differences.

The connection between food insecurity and adiposity is likely complex, involving biological and behavioural factors. Adolescents facing food insecurity often have limited access to nutrient‐rich foods, resulting in reliance on higher energy‐dense, low‐nutrient options contributing to greater adiposity [43, 44, 45]. Furthermore, the psychological stress linked to food insecurity can affect eating habits and metabolic functions, leading to increased weight gain [46, 47, 48, 49]. Recent research indicates that food insecurity might also disrupt sleep patterns [50] and decrease physical activity [51], which influences weight gain.

A particular strength of our study is its longitudinal design, which allowed us to examine how food insecurity affects changes in adiposity over a 2‐year period during adolescence. Given the lack of longitudinal studies examining the effect of food insecurity on adolescent adiposity [15], our findings provide an important contribution to this topic. Furthermore, this study expands investigation beyond BMI with the inclusion of additional comprehensive objective measures of adiposity. Lastly, our inclusion of potential confounders, like poverty, lends strength to our observation that food insecurity appears to be an important contributor to adiposity in adolescents. While our study offers significant insights, some limitations should also be noted. Our assessment of food insecurity relied on parental self‐reporting, which may have been influenced by recall bias or a desire to present oneself favorably. Furthermore, because food insecurity is dynamic and episodic, we are limited by the study's measurement of food insecurity at only two time points and our analysis of only baseline food insecurity. More frequent, repeated measures of food insecurity and outcomes would better capture these relationships. Additionally, our sample's relatively small number of food‐insecure participants may have reduced the statistical power to identify differences between boys and girls.

Our results can inform public health and policy. Given the strong links between food insecurity and increased accumulation of body fat, initiatives aimed at reducing food insecurity could be essential in preventing obesity in adolescents. For example, policies that enhance access to nutritious foods, like expanding Supplemental Nutrition Assistance Program (SNAP) benefits or launching school nutrition programs, may mitigate the adverse effects of food insecurity on adolescent health. Additional help for families to access and utilise these resources may be needed to realize the full benefit of nutrition assistance. Programs under the ‘Food Is Medicine’ initiative also reduce food insecurity, yet they are not yet widely implemented. Additionally, interventions designed to reduce unhealthy weight gain during adolescence may require tailoring for food‐insecure households.

In conclusion, this study found that food insecurity is associated with greater increases in adolescent adiposity over a 2‐year period. These findings underscore the critical need for obesity prevention efforts for adolescents. Integrating food insecurity screening into pediatric care, developing targeted behavioral interventions that account for the context of food insecurity, and strengthening nutrition assistance policies may help mitigate excess weight gain among vulnerable youth. Future research should explore underlying mechanisms linking food insecurity to adiposity in adolescents.

## Funding

This research was supported by the United States Department of Agriculture (3092‐51000‐056‐04A; ClinicalTrials.gov: NCT02784509, PI: Amanda E. Staiano), and the National Institutes of Health (P30DK072476, PI: Eric Ravussin; U54 GM104940, PI: John Kirwan).

## Conflicts of Interest

The authors declare no conflicts of interest.

## Supporting information


**Table S1:** Results of sensitivity analyses, compared to Table 3 results, for the effect of food insecurity on 2‐year changes in adolescent adiposity.

## Data Availability

The data that support the findings of this study are available on request from the corresponding author. The data are not publicly available due to privacy or ethical restrictions.
