# Supplementary material for: Household Food Insecurity Is Associated With Higher Adiposity Over Time Among Adolescents in Louisiana
Source: Pediatr Obes. 2026 Feb 5;21(2):e70084. doi: 10.1111/ijpo.70084 (PMC12874501; doi:10.1111/ijpo.70084)
Supplement: Supplementary file 1 — Table S1: Results of sensitivity analyses, compared to Table 3 results, for the effect of food insecurity on 2‐year changes in adolescent adiposity. [file IJPO-21-e70084-s001.pdf]

Household Food Insecurity is Associated with Higher Adiposity Over Time among Adolescents in Louisiana

Ashley Fenton, Ph.D.[1]
Amanda E. Staiano, Ph.D.[2]
Michael Celestin, Ph.D.[1]
Tekeda Ferguson, Ph.D.[1]
Candice A. Myers, Ph.D.[2]
Tung-Sung Tseng, DrPH[1]
Stephanie T. Broyles, Ph.D.[1,2]

[1]Louisiana State University Health Sciences Center, School of Public Health
[2]Pennington Biomedical Research Center, Baton Rouge, Louisiana

Table S1. Results of sensitivity analyses, compared to Table 3 results, for the effect of food insecurity on two-year changes in adolescent adiposity.

Model	Table 3	Sens Analysis 1	Sens Analysis 2
**Imputation**	None	None	All missing data for ppts with baseline & follow-up visits
**Food Insecurity**	Baseline	Pooled	Baseline
**N**	222	220	258
**Outcome**	**Beta (p-value)**	**Beta (p-value)**	**Beta (p-value)**
BMI_{p95}, %	6.0 (p=0.0082)	5.6 (p=0.0063)	5.9 (p=0.0075)
WC, cm	4.1 (p=0.0158)	4.2 (0.0054)	3.5 (p=0.0386)
TBF, %	3.0 (p=0.0194)	2.5 (0.0304	2.9 (p=0.0172)
VAT, kg	0.16 (p=0.0163)	0.12 (0.0420)	0.14 (p=0.0126)

Abbreviations: BMI_{p95}: percentage of the 95th BMI percentile; WC: Waist circumference, TBF: Total Body Fat percentage, VAT: Abdominal visceral adipose tissue

Corresponding Author:
Stephanie Broyles
Pennington Biomedical Research Center
6400 Perkins Road
Baton Rouge, LA 70808
Telephone: 225-763-2760
Fax: 225-763-2760
Email: stephanie.broyles@pbrc.edu
